# Deriving Optimized PID Parameters of Nano-Ag Colloid Prepared by Electrical Spark Discharge Method

**DOI:** 10.3390/nano10061091

**Published:** 2020-06-01

**Authors:** Kuo-Hsiung Tseng, Yur-Shan Lin, Yun-Chung Lin, Der-Chi Tien, Leszek Stobinski

**Affiliations:** 1 Department of Electrical Engineering, National Taipei University of Technology, Taipei 10608, Taiwan; linyurshan@gmail.com (Y.-S.L.); tienderchi@gmail.com (D.-C.T.); 2 Power Department, Quanta Computer lnc., Taipei 111, Taiwan; jklook355357@gmail.com; 3 Materials Chemistry, Warsaw University of Technology, Warynskiego 1, 00-645 Warsaw, Poland; L.Stobinski@ichip.pw.edu.pl

**Keywords:** electrical spark discharge method, nano-Ag colloid, Ziegler–Nichols method, electrical discharge condition, short circuits

## Abstract

Using the electrical spark discharge method, this study prepared a nano-Ag colloid using self-developed, microelectrical discharge machining equipment. Requiring no additional surfactant, the approach in question can be used at the ambient temperature and pressure. Moreover, this novel physical method of preparation produced no chemical pollution. This study conducted an in-depth investigation to establish the following electrical discharge conditions: gap electrical discharge, short circuits, and open circuits. Short circuits affect system lifespan and cause electrode consumption, resulting in large, non-nanoscale particles. Accordingly, in this study, research for and design of a new logic judgment circuit set was used to determine the short-circuit rate. The Ziegler–Nichols proportional–integral–derivative (PID) method was then adopted to find optimal PID values for reducing the ratio between short-circuit and discharge rates of the system. The particle size, zeta potential, and ultraviolet spectrum of the nano-Ag colloid prepared using the aforementioned method were also analyzed with nanoanalysis equipment. Lastly, the characteristics of nanosized particles were analyzed with a transmission electron microscope. This study found that the lowest ratio between short-circuit rates was obtained (1.77%) when PID parameters were such that K_p_ was 0.96, K_i_ was 5.760576, and K_d_ was 0.039996. For the nano-Ag colloid prepared using the aforementioned PID parameters, the particle size was 3.409 nm, zeta potential was approximately −46.8 mV, absorbance was approximately 0.26, and surface plasmon resonance was 390 nm. Therefore, this study demonstrated that reducing the short-circuit rate can substantially enhance the effectiveness of the preparation and produce an optimal nano-Ag colloid.

## 1. Introduction

Nanotechnology entails the science and technology that apply the physical and chemical characteristics of substances smaller than 100 nm [1] to the design and production of new components and systems. Because of the structural characteristics of increased surface area and a lack of periodic regularity, as well as interactions between the shape and surface size of nanostructures [2], nanosized substances display physical, chemical, and biological characteristics that are distinct from those of other substances [3]. The techniques used to prepare nanoparticles can be classified into chemical methods and physical methods. Nearly all the chemical methods require the addition of surfactants. The physical methods include mechanical milling [4], thermal evaporation [5], the submerged arc nanoparticle synthesis system (SANSS) [6], and so on. Mechanical milling has the potential for contamination from the balls and the atmosphere. Thermal evaporation has the drawback of containing the carrier gas and catalytic particle in the grown nanostructure. The process of SANSS should occur within a vacuum chamber. In this work, silver nanoparticles with good purity were prepared at a normal temperature and pressure using the electrical spark discharge method (ESDM).

Electrical discharge machining (EDM) is used for a wide variety of materials because it uses heat rather than mechanical principles. For example, the electric spark discharge method (ESDM) has been applied on hard materials, small components, and components requiring high-precision machining or a complex shape. Two primary targets of ESDM are precision and refinement, and the major ESDM trends are powder-mixed EDM, precision wire EDM, and a hybrid of microelectrical discharge machining (micro-EDM) and polishing [7].

**Figure 1:**
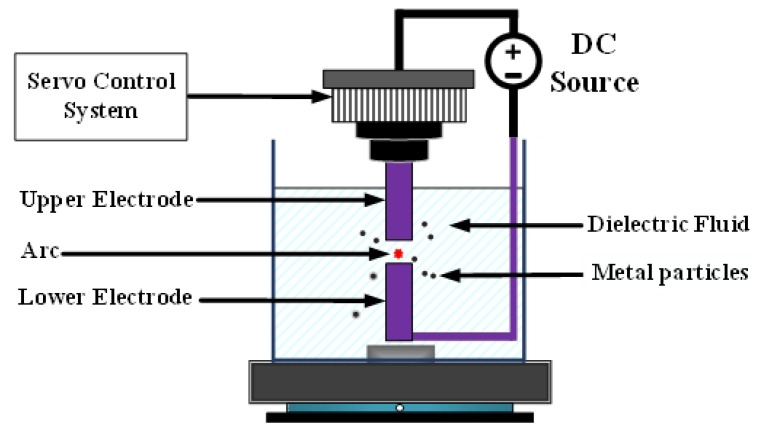
Electrical discharge machining.

Figure 1 depicts EDM [8]. Both the upper and lower electrodes were connected to the metal part of the workpiece and immersed in a highly insulating dielectric liquid. In nanotechnology research, deionized water is often used as the dielectric liquid. Application of direct current voltage enables the upper electrode to be controlled by the servo control system and to move slowly toward the lower electrode. Because the two ends of the workpiece are not in direct contact, no physical force, such as contact or cutting, is involved. When the distance between the two electrodes reaches approximately 30 μm, a discharge column is formed between the two electrodes [9], generating the so-called spark. The electric arc can reach temperatures as high as 5000–6000 K [10], causing atoms at the metal surface to melt. This method is called the ESDM.

Applying ESDM in combination with EDM, the research team of this study successfully prepared several kinds of nanocolloid, including nano-Au, nano-TiO_2_, nano-Al, and nanographene colloids [11,12,13,14]. Because traditional EDM equipment is outdated and difficult to maintain, micro-EDM equipment was developed. Micro-EDM works on the same principle as EDM. The analysis of nano-Au and nano-Ag colloids prepared with this equipment, using high-precision instruments, showed that all of the colloidal particles displayed nanoparticle characteristics [15]. Studies of nanometal colloids prepared using micro-EDM have taken into account only the discharge success rate. This study observed the conditions for currents flowing between electrodes, including gap electrical discharge and electrode short circuits, and explored these phenomena in depth. Because the short-circuit phenomenon may exert an adverse effect on equipment, this study designed a set of logic judgment circuits to identify short circuits. The short-circuit rate was calculated by computer software using the aforementioned circuit output signals. In addition, this study used the Ziegler–Nichols classic proportional–integral–derivative (PID) method to determine optimal PID parameters to reduce the short-circuit rate. Lastly, how short-circuit rate is related to particle analysis, zeta potential, and absorbance was explored [16,17,18,19].

## 2. Materials and Methods 

### 2.1. Logic Circuit Design

**Figure 2:**
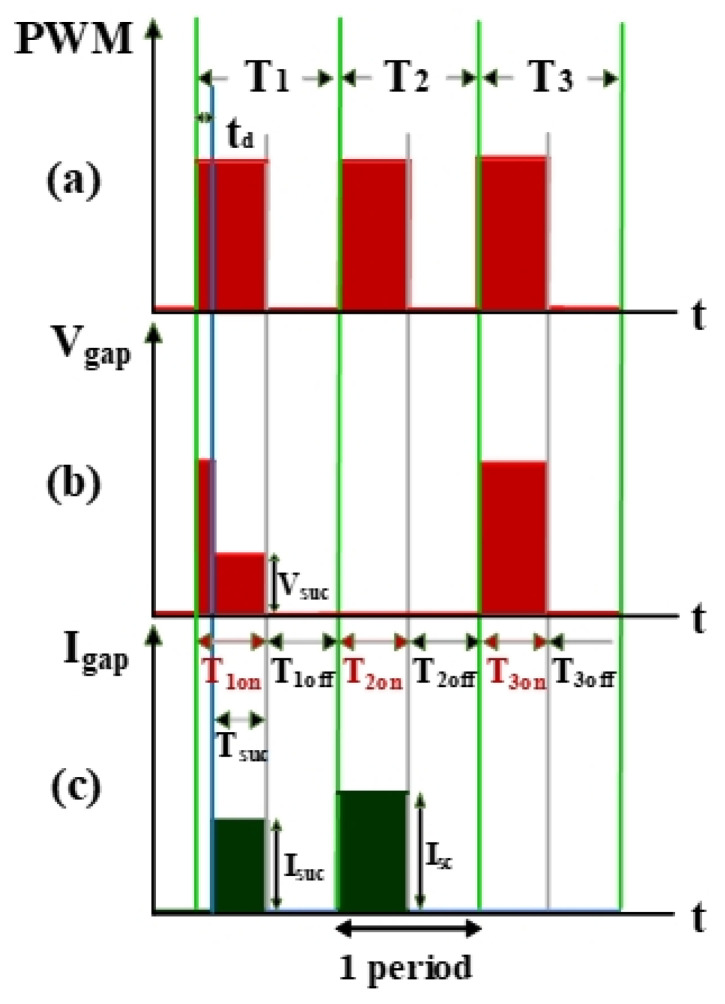
Waveforms of (**a**) pulse width modulation (PWM), (**b**) V_gap_, and (**c**) I_gap._

The electrical discharge conditions of pulse width modulation (PWM), gap voltage (V_gap_), and gap current (I_gap_) during electrical discharge are shown in Figure 2. This comprises the transistor-on time (T_on_) and the transistor-off time (T_off_). T_suc_ represents the successful electrical discharge time. T1 represents the time period for gap electrical discharge. The dielectric fluid was observed to exhibit an insulation breakdown after the spark time lag (t_d_); V_gap_ also decreased to V_suc_, and I_gap_ increased to I_suc_. T2 is the time period for short circuits, indicating the time at which an electrode short circuit occurred because the anode and the cathode were too close to each other. V_gap_ decreased to 0, and I_gap_ increased to I_sc_. The T2 time period is for the short-circuit condition. It indicates the time for open circuits at which the electric field in the electrode gap was insufficiently strong to break the insulation of the dielectric fluid. V_gap_ shows an open-circuit condition when the value for I_gap_ remained at 0 A [20].

This study’s major contribution is the identification of three electrical discharge conditions: gap electrical discharge, short circuits, and open circuits. The logic circuits were designed to identify all electrical discharge conditions. First, a set of logic circuits identifying and differentiating between gap electrical discharge and short circuits was designed. The circuits first acquire V_gap_ and I_gap_, and these signals are then processed by the comparator and the AND gate (The AND gate is a digital logic gate that implements logical conjunction) to generate signals determining successful electrode gap discharge. The successful discharge signals are then compared with the counter pulse signals from the computer end to obtain the number of instances of successful electrical discharge. The short-circuit rate is determined by directly comparing the counter pulse signal and the I_gap_ signal output by the computer. When the system’s electrical discharge is successful, V_gap_ decreases and I_gap_ increases [21]. Details of the simulation process are presented as follows:

#### 2.1.1. V_gap_ High/Low Levels and I_gap_ Simulation

**Figure 3:**
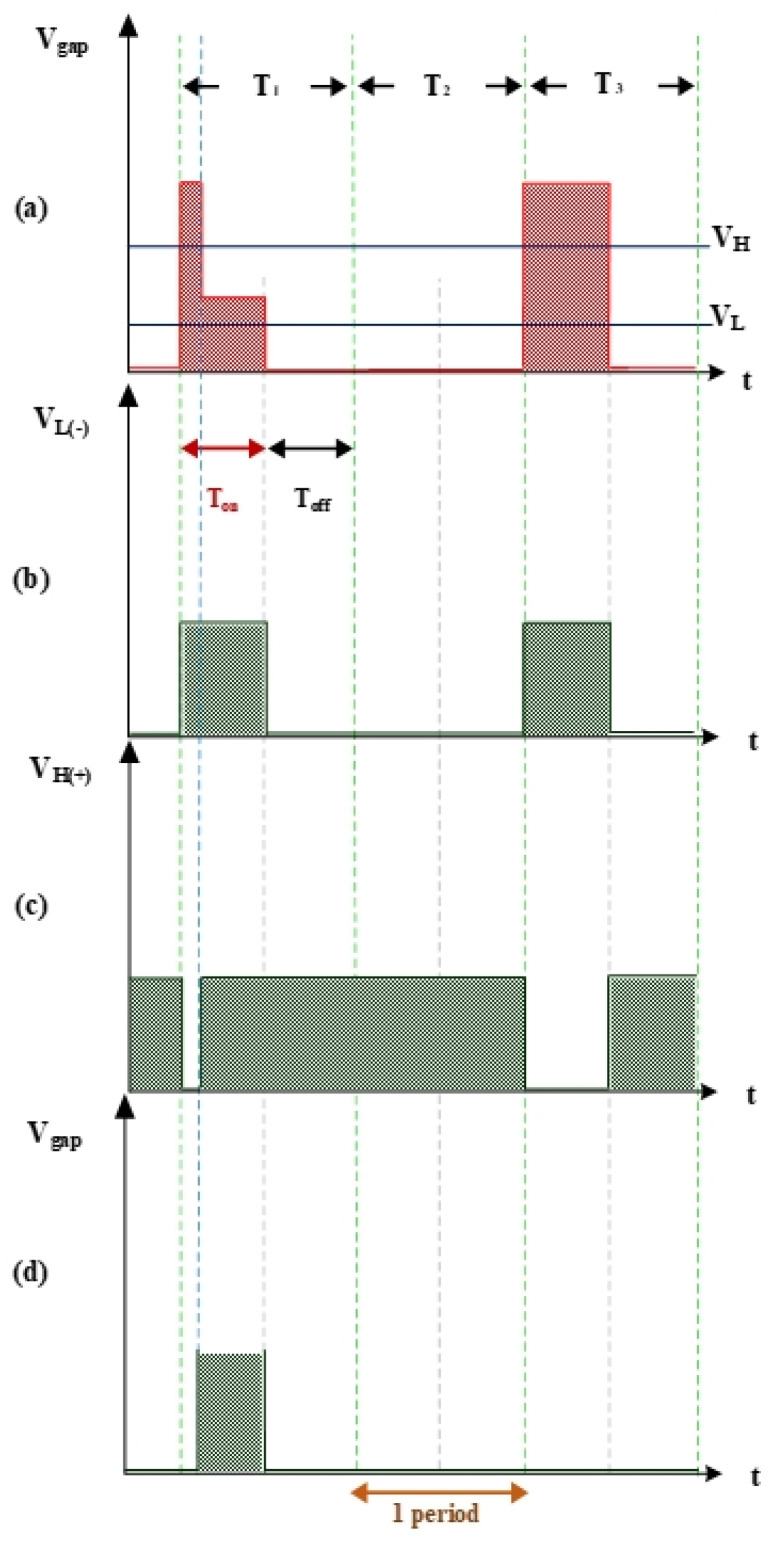
Voltage waveforms: (**a**) V_gap_, V_H_, and V_L_; (**b**) V_L_ input into the comparator; (**c**) V_H_ input into the comparator; and (**d**) gap electrical discharge.

**Figure 4:**
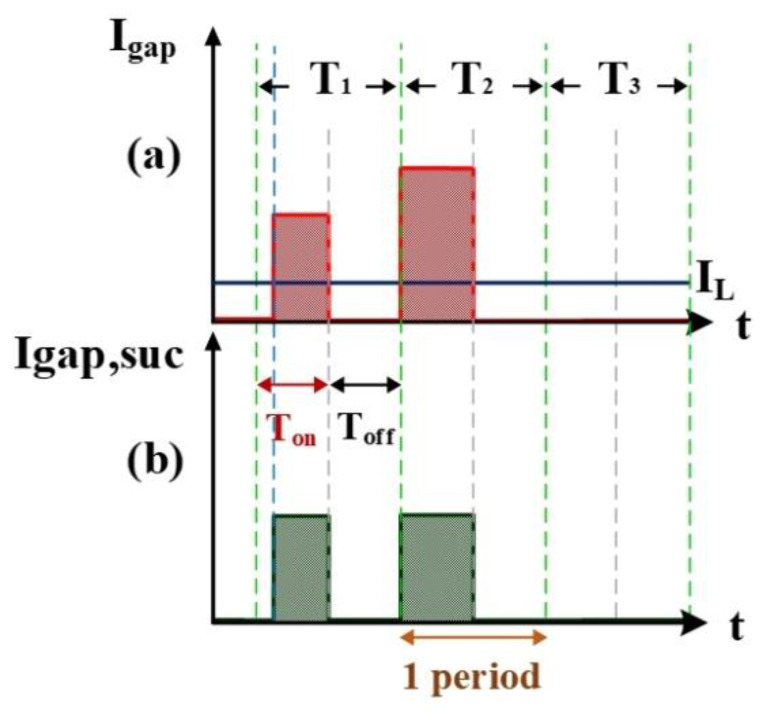
Current I_gap_: (**a**) I_gap_ and I_L_, and (**b**) I_gap_ and I_L_ input into the comparator.

**Figure 5:**
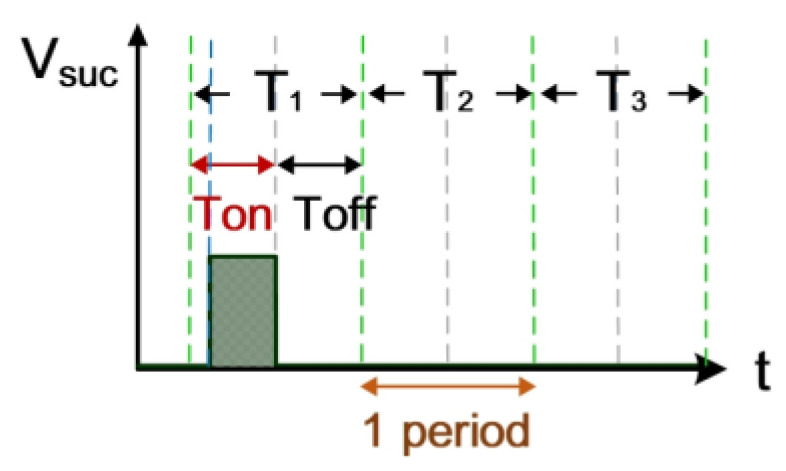
Gap electrical discharge simulation.

Figure 3 shows the measurement of electrode gap voltage conditions. T1 is the time period of the electrical discharge condition. T2 is the short-circuit condition. T3 is the open-circuit condition. Figure 3a illustrates the V_gap_ waveform. V_L_ is the low-level voltage of V_gap_ for successful electrical discharge, whereas V_H_ is the high-level voltage. Figure 3b is the simulated waveform of the comparison between V_gap_ and V_L_ by a comparator. When V_gap_ is greater than V_L_, the comparator output signal V_L(-)_ indicates a high potential. Figure 3c is the simulated waveform of the comparison between V_gap_ and V_H_ by a comparator. When V_gap_ is smaller than V_H_, the comparator output signal V_H(+)_ indicates a high potential. Figure 3d shows the logic operation result for V_L(-)_ and V_H(+)_ through the AND gate. Output signals for V_gap_ and V_suc_ are 1 only when both V_L(-)_ and V_H(+)_ are 1. Figure 4 illustrates the identification of the electrode gap current conditions. Figure 4a is the I_gap_ waveform. The low-level current of I_gap_ for successful electrical discharge is I_L_. Figure 4b shows the simulated waveform of the comparison between I_gap_ and I_L_ by a comparator. When I_gap_ is greater than I_L_, comparator output signals I_gap_ and I_suc_ indicate a high potential. Figure 5 indicates the logic operation result of I_gap,suc_ and V_gap,suc_ through the AND gate. The output signal for V_suc_ is 1 only when both I_gap,suc_ and V_gap,suc_ are 1.

#### 2.1.2. Computation Simulation of Electrical Discharge Success Rate

**Figure 6:**
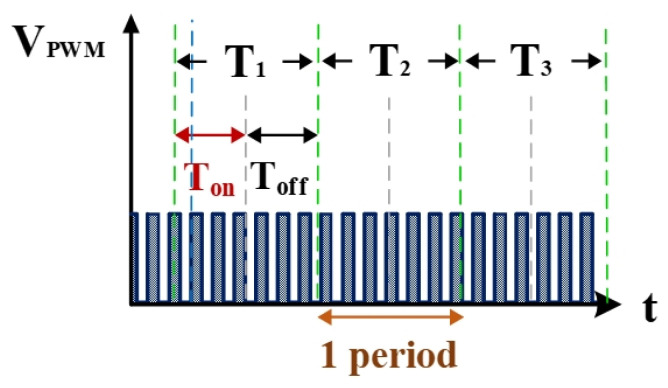
Quantified signals.

**Figure 7:**
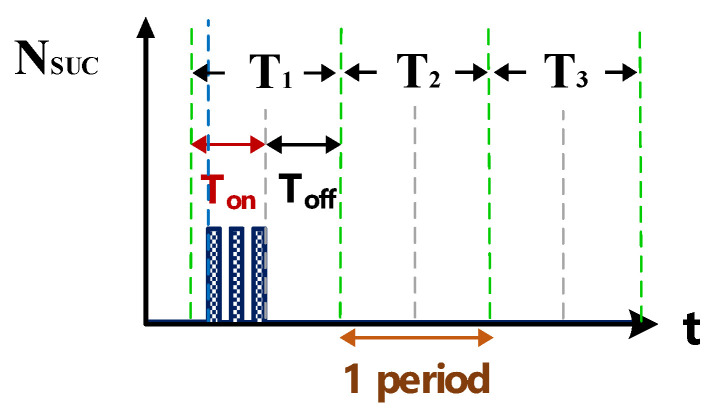
Counter waveform of discharge success rate.

Figure 6 shows the counter pulse signal V_PWM_ output by the computer (with a frequency of 1 MHz and a duty cycle of 0.5). This signal was generated by the RT-DAC4/PCI interface card controlled by the software VisSim at the computer end. This signal constitutes the time base of the hardware circuits. Figure 7 shows the counter waveform signal N_SUC_ of successful electrical discharge. This waveform is the result of the computation of the signals for V_suc_ (Figure 5) and V_PWM_ (Figure 6) passing through the AND gate. Using the signal N_SUC_, the VisSim software at the computer end can count the number of accumulated successful electrical discharges.

#### 2.1.3. Computation Simulation of Short-Circuit Rate

**Figure 8:**
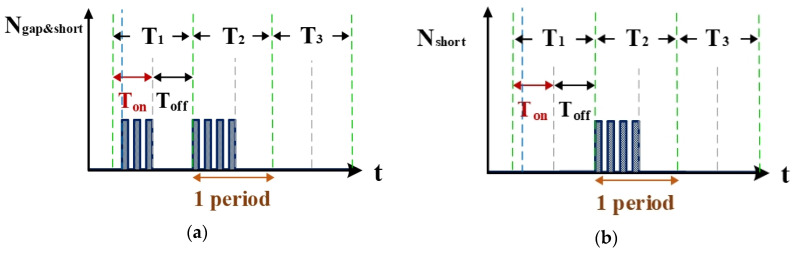
Counter waveforms of (**a**) signal I_gap_ and (**b**) short circuits.

Figure 8a is the counter waveform of signal N_gap&short_. This signal is the computation result for the signals I_gap,suc_ (Figure 4b and V_PWM_ (Figure 7) passing through the AND gate. According to the signal for N_SUC_, the VisSim software at the computer end can count the total numbers of gap electrical discharge and short-circuit instances. Figure 8b shows the signal for N_short_, which indicates the number of short circuits counted. This signal is the difference between the waveforms of N_SUC_ (Figure 7) and N_gap&short_ (Figure 8a). According to the aforementioned logic judgment simulation and the condition judgment mechanism, a set of optimized logic judgment circuits was designed. The functions of this circuit set encompass computation of the electrical discharge success rate, the short-circuit rate, and the open-circuit rate.

### 2.2. Electrical Discharge Process Optimization

**Figure 9:**
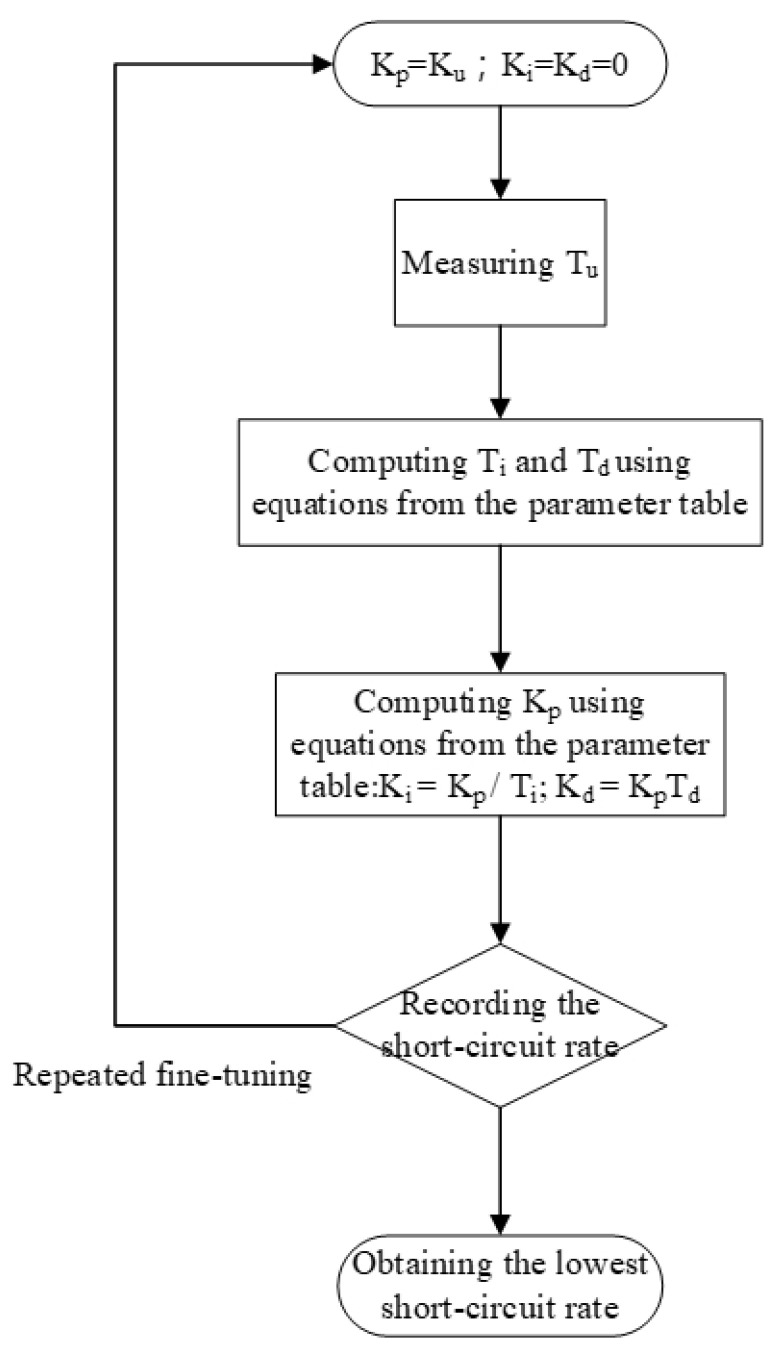
Proportional–integral–derivative (PID) parameter fine-tuning flow chart.

This study primarily explored the application of PID closed-circuit controllers in EDM for controlling electrical discharge gap voltage. The key objective is to determine the short-circuit rate and to use the Ziegler–Nichols method PID to effectively reduce the short-circuit rate [22,23,24]. In this study, an Ag wire of 99.9% purity with 1 mm and 2 mm diameter was used as the metallic material for the anode and cathode electrode, respectively. The dielectric fluid was low-conductivity deionized water. The electrical discharge voltage was 100 V, and T_on_ and T_off_ were set at 10 µs. The process time was 120 s at ambient temperature (25 °C) and atmospheric pressure (1atm). The preparation capacity was 150 mL. Table 1 shows the control parameters of the Ziegler–Nichols method. Table 2 shows the process parameters. PID parameters were fine-tuned to reduce the short-circuit rate, and K_u_ was the critical gain of the PID controller. The micro-EDM motor does not function when K_u_ < 1.25, whereas the periodic waveforms are unstable and nonsine waves when K_u_ > 2.5, resulting in divergence. To avoid said problems, K_u_ was set to between 1.25 and 2.5. On-machine fine-tuning was used, and the short-circuit rate was recorded for selecting optimal PID parameters for the micro-EDM. Professional instruments were used to analyze the absorbance, particle size, and zeta potential of the nano-Ag colloid. The relationship between the characteristics of the colloid and the short-circuit rate was also examined. Figure 9 shows the fine-tuning process for PID parameters.

## 3. Results

### 3.1. PID Fine-Tuning Optimization

**Figure 10:**
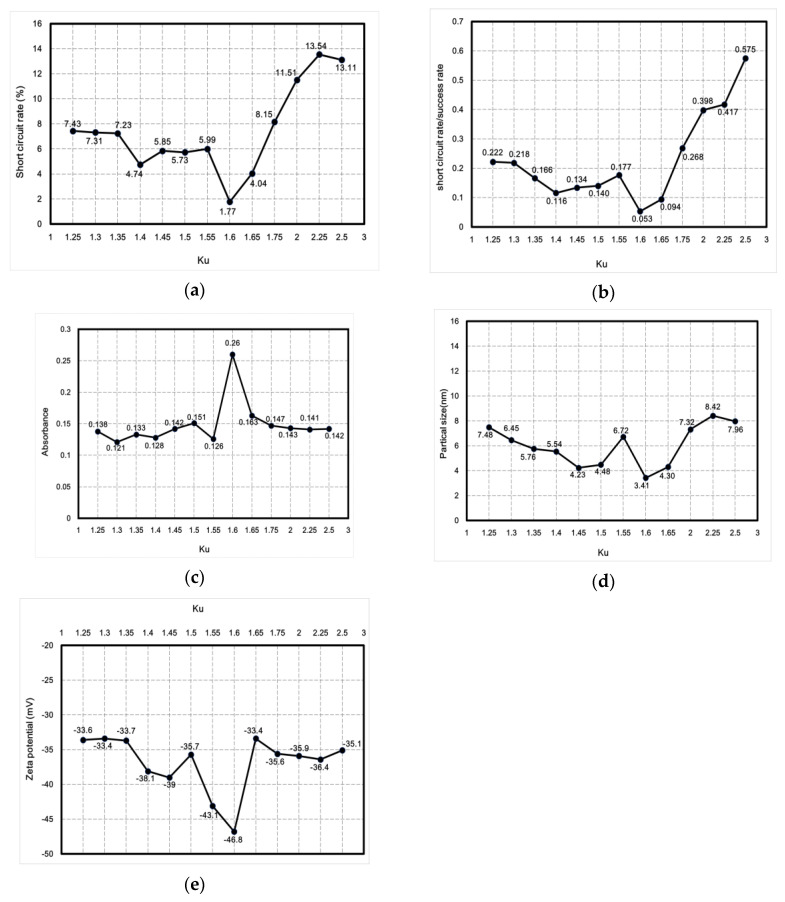
Curves describing the relationship between K_u_ and nanoparticle characteristics: (**a**) short-circuit rate, (**b**) ratio between short-circuit rate and discharge success rate, (**c**) absorbance, (**d**) particle size, and (**e**) zeta potential.

This study used the Ziegler–Nichols method for fine-tuning PID parameters to reduce the short-circuit rate. One advantage of the method in question is that proper tuning can be achieved according to changes in the parameters. The PID fine-tuning process parameter K_u_ was between 1.25 and 2.5, and tuning was performed in an interval of 0.25. After tuning, the short-circuit rate, absorbance, particle size, zeta potential, and ratio between the short-circuit rate and the discharge success rate were analyzed and compared using professional instruments to determine the best parameters for micro-EDM. Figure 10 shows the curve describing the relationship between K_u_ and nanoparticle characteristics. Figure 10a shows the curve describing the relationship between K_u_ and the short-circuit rate. Figure 10b shows the curve describing the relationship between K_u_ and the ratio between the short-circuit rate and the discharge success rate. Figure 10c shows the curve describing the relationship between K_u_ and absorbance. Figure 10d shows the curve describing the relationship between K_u_ and particle size. Figure 10e shows the curve depicting the relationship between K_u_ and zeta potential. The curves demonstrate that when K_u_ was 1.6, the lowest short-circuit rate (1.77%) was obtained using PID parameters (i.e., K_p_ = 0.96; K_i_ = 5.760576; K_d_ = 0.039996), with the ratio between the short-circuit rate and the discharge success rate being 0.053. Moreover, the nano-Ag colloid had an absorbance of 0.26, a zeta potential of −46.8 mV, and a particle size of 3.41 nm. The values of these nanoparticle characteristics were optimal. The data demonstrated that all nanoparticle-related characteristics were optimal when the short-circuit rate was the lowest. Therefore, effectively reducing the short-circuit rate enhances the overall electrical discharge effect of micro-EDM.

### 3.2. Nano-Ag Colloid Characteristics Analysis

Applying micro-EDM, this study prepared a nano-Ag colloid with a minimum short-circuit rate using optimal PID parameters (K_p_ = 0.96, K_i_ = 5.760576, K_d_ = 0.039996, and therefore K_u_ = 1.6). By analyzing the colloid with professional instruments, this study demonstrated that the specimen is nanosized. Details are presented in the following.

#### 3.2.1. UV-Vis and Zeta Potential Analysis

**Figure 11:**
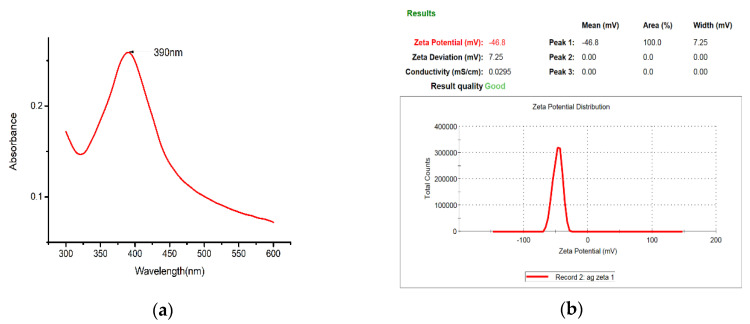
Nano-Ag colloid analysis results for (**a**) UV-Vis and (**b**) zeta potential.

The nano-Ag colloid was analyzed with a spectrophotometer (UV-Vis), which demonstrates the relationship between absorbance and wavelength. Various studies have defined the characteristic wavelength of nano-Ag colloids to be between 380 and 410 nm (Figure 11a). The analysis here yielded a characteristic wavelength of 390 nm for the nano-Ag colloid prepared using ESDM, a nanosized characteristic. The absorbance of the nano-Ag colloid was 0.26, and the value of absorbance of the colloid was mostly proportional to the concentration of the colloid. In this study, the zeta potential of nanoparticles was measured with a Zetasizer. When the absolute value of the potential was greater than 30 mV, the metal particles in the colloid exhibited high suspension stability. The analysis result for zeta potential is shown in Figure 11b. The average value for zeta potential measured was −46.8 mV, indicating that the nanocolloid prepared here exhibited desirable suspension stability. 

#### 3.2.2. Transmission Electron Microscopy and Energy-Dispersive X-ray Spectroscopy 

**Figure 12:**
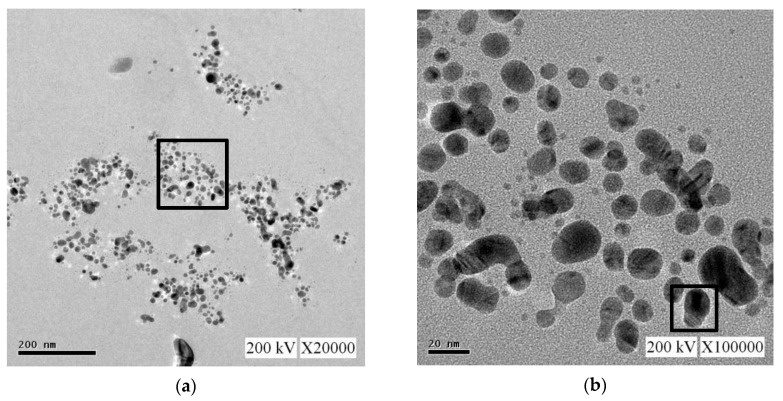

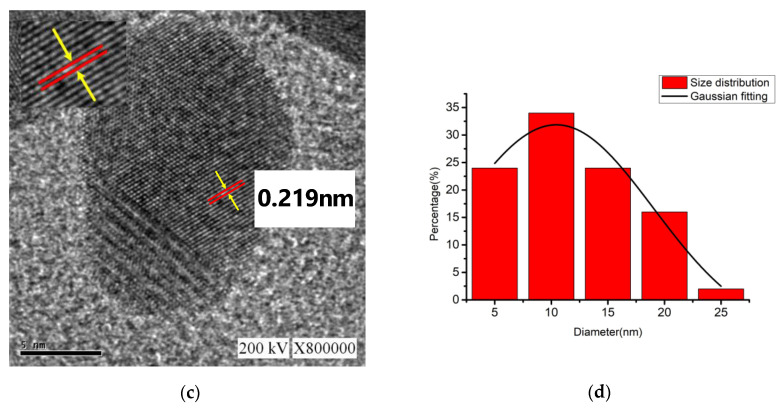
Nano-Ag TEM images: (**a**) 200 nm; (**b**) 20 nm; (**c**) 5 nm; and (**d**) size distribution.

**Figure 13:**
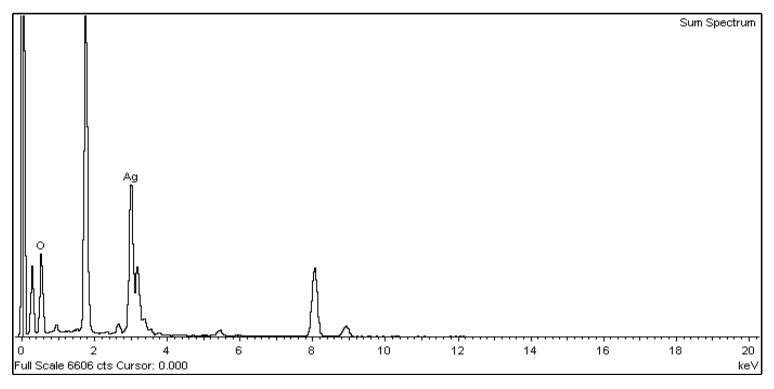
Nano-Ag particle energy-dispersive X-ray spectroscopy (EDS) analysis.

For the nano-Ag colloid prepared using ESDM, nano-Ag particles were observed microscopically with a transmission electron microscope. Figure 12a shows a nano-Ag colloidal structure at a scale of 200 nm. Figure 12b is the enlarged image (at a scale of 20 nm) of the image inside the box in Figure 12a. Figure 12c is the enlarged image (at a scale of 5 nm) of the image inside the box in Figure 12b. The spacing between lattice lines (d-spacing) was 0.219 nm. Figure 12d is obtained from the results of TEM (Figure 12b), which shows the size distribution of nano-Ag participles. According to the figure, 0–5 nm nano-Ag particles accounted for 24% of all nano-Ag particles, 6–10 nm for 34%, 11–15 nm for 24%, 16–20 nm for 16%, and 21 nm or more for 2%. This finding showed that most of the nano-Ag particles had sizes of 6–10 nm. Energy-dispersive X-ray spectroscopy (EDS) is a type of analytical technology which uses the characteristic X-rays of electron beams to analyze the chemical composition of specimens when each element has distinct spectral characteristics. Figure 13 shows the graph of EDS analysis of the nano-Ag colloid. The result indicates that the nano-Ag colloid prepared using ESDM contained only two elements: oxygen (O) and silver (Ag). 

## 4. Conclusions

Using real-time micro-EDM monitoring, this study designed a logic judgment circuit set to determine all EDM conditions, including gap electrical discharge, open circuits, and short circuits of the EDM equipment. Signals were sent to the software end to determine the probability for each of the aforementioned conditions. The result demonstrated that short circuits may adversely affect equipment, and reducing the short-circuit rate by PID tuning therefore improves the overall electrical discharge effect. Lastly, the results of an analysis using high-precision instruments, including UV-Vis and the Zetasizer, indicated the existence of nano-Ag particles in the deionized water. Moreover, when the short-circuit rate was the lowest, all nanoparticle characteristics were most optimal. This study makes the following contributions:

1. In this study, a nano-Ag colloid was prepared using the electric spark discharge method (ESDM) with electrode material (Ag with 99.9% purity) in 150 mL of deionized water. The diameter of the anode electrode was 1 mm, and the diameter of the cathode electrode was 2 mm. With the electrical discharge voltage = 100 V, duty cycle (Ton-Toff) = 10-10 μs, and a process time of 120 s, silver nanoparticles with an absorbance of 0.26 could be prepared.
2. The study used self-developed micro-EDM and ESDM to prepare a nano-Ag colloid. This method requires no additional surfactant or other chemical materials and can be used at the ambient temperature and pressure. Moreover, this novel physical method for preparing nano-Ag colloids results in no chemical pollution.
3. When using ESDM to prepare a nano-Ag colloid, the electrical discharge conditions were observed to comprise gap electrical discharge, short circuits, and open circuits. The short-circuit phenomenon was explored in depth in this study, and a self-developed logic judgment circuit set was applied to identify short circuits. Signals were then sent to computer software for computation of the rate. Short circuits may have exerted an adverse effect on the equipment.
4. This study showed that PID parameters such that K_p_ was 0.96, K_i_ was 5.760576, and K_d_ was 0.039996 (and, as a result, K_u_ was 1.6) produced optimal values for absorbance (0.26), surface plasmon resonance (390 nm), zeta potential (−46.8 mV), particle size (3.41 nm), short-circuit rate (1.77%), and the ratio between the short-circuit rate and the discharge success rate (0.053). Therefore, this study demonstrated that the lower the short-circuit rate is, the more the nanocharacteristics are optimized.

## Figures and Tables

**Table 1:** Control parameters of the Ziegler–Nichols method.

	K_p_	T_i_	T_d_
Ziegler–Nichols method	0.6 × K_u_	T_u_/2	T_u_/8
K_u_ = critical gain, T_u_ = period of oscillation, K_i_ = K_p_/T_i_, K_d_ = K_p_ × T_d_			

**Table 2:** Process parameters.

Title	Parameters	Title	Parameters
Ambient temperature	25 °C	Atmospheric pressure	1 atm
Electrical Discharge voltage	100 V	Electrical discharge current	4 A
Duty cycle	T_on_-T_off_ 10-10 (μs)	Electrode material (purity)	Ag (99.99%)
Process time	120 s	Dielectric fluid	Deionized water
Electrode diameter	Anode 1 mm Cathode 2 mm	Preparation capacity	150 mL
